# Characterization and Synergistic Antimicrobial Evaluation of Lipopeptides from *Bacillus amyloliquefaciens* Isolated from Oil-Contaminated Soil

**DOI:** 10.1155/2019/3704198

**Published:** 2019-03-10

**Authors:** Pratiwi Sudarmono, Ahmad Wibisana, Lira W. Listriyani, Saleha Sungkar

**Affiliations:** ^1^Department of Microbiology, Faculty of Medicine, Universitas Indonesia, Jakarta, Indonesia; ^2^Biotech Center, Agency for the Assessment and Application of Technology, Jakarta, Indonesia; ^3^Department of Parasitology, Faculty of Medicine, Universitas Indonesia, Jakarta, Indonesia

## Abstract

Lipopeptides show great potential for biomedical application. Several lipopeptides exhibit narrow and broad-spectrum inhibition activities. The aim of the study is to characterize the lipopeptides produced by *B. amyloliquefaciens* strain MD4-12 and evaluate the synergistic antimicrobial activity in combination with a conventional antibiotic against Gram-negative bacteria. *B. amyloliquefaciens* strain MD4-12 was isolated from oil-contaminated soil. The isolate was cultivated in McKeen medium, and the lipopeptides were isolated by precipitation and extraction with methanol. Characterization of the lipopeptides by ESI-MS gave nine mass ion peaks with *m*/*z* 994–1072, resulted from protonating of the main ions in [M + H]^+^ and [M + Na]^+^ ion form. These mass ion peaks attributed to surfactin homologs. By tandem mass spectrometry, five variants of surfactin with the same amino acid sequence in peptide moiety could be revealed. The peptide moiety contains seven amino acids identified as Glu-Leu/Ile-Leu-Val-Asp-Leu-Leu/Ile while the fatty acid moiety comprises a different length of chain from C_12_ to C_16_. Surfactin showed antibacterial activity against various Gram-positive and Gram-negative bacteria. Combination surfactin with ampicillin showed a synergistic effect against *P. aeruginosa* ATCC 27853.

## 1. Introduction

Lipopeptides (LPs) are small molecules consisting of a linear or cyclic peptide linked with a lipid tail or other lipophilic molecules [1]. Lipopeptides are produced by several groups of bacteria, both Gram-positive and Gram-negative bacteria. The high diversity in chemical structure and LPs-producing microorganisms suggests that the LPs compounds may serve different and possibly multiple purposes. Polymyxin A is the example of lipopeptide with an antimicrobial activity which isolated from the soil bacterium *Bacillus polymyxa* [2]. Recently, several lipopeptides with diverse activities have been characterized, namely, as cytotoxic [3], antibacterial [4, 5], antifungal [6, 7], antiviral [8, 9], plant pathogenicity [10, 11], and anticancer [12, 13]. Some of them have been commercially used as an antibacterial drug such as daptomycin [14], caspofungin [15], micafungin [16], vancomycin, and teicoplanin [17].

The bacilli are known for their ability to produce several lipopeptides with potential application to clinical and biocontrol purposes. In term of their biosynthetic pathway, lipopeptides can be grouped into two classes. The first class consists of peptides synthesized through ribosomal pathways, and the second class consists of nonribosomal peptides. The metabolites included in the first class consist of bacteriocins and bacteriocin-like inhibitory substances, for examples, subtilin, nisin, ericins, mersacidin, subtilosin A, sublancin 168, coagulin, and lichenin, while the metabolites included in the second class, nonribosomal synthesized peptides, include cyclic lipopeptides.

The cyclic lipopeptides produced by *Bacillus* consist of three families, classified as surfactin, iturin, and fengycin. Each family has different variants with the same peptide length but with different amino acid composition. Moreover, each variant can have several homologs with different length and isomer of the fatty acid chain, leading to a remarkable structural diversity [18]. The diversity of lipopeptide chemical structures and characteristics are influenced by producer strain and ecological differences of microbial producers [19].

In this study, *Bacillus* isolated from oil-contaminated soil was used to produce lipopeptides via a fermentation process. The lipopeptides were purified and characterized for their structures using mass spectrometry. The synergistic combination with conventional antibiotics was evaluated against Gram-negative bacteria.

## 2. Materials and Methods

### 2.1. Microorganisms, Production Medium, and Culture Condition

The lipopeptides producer was isolated from an oil-contaminated soil sample collected from the area of Pertamina refinery oil plant in Palembang, Indonesia. Identification of the strain was conducted by analyzing of 16S rRNA gene sequence. The 16S rRNA gene was amplified by PCR using universal primers of 8F and 1492R. PCR products were sequenced by Applied Biosystems 3130XL using 765F and 1441R primers and aligned by CLUSTAL_X program of EBI version 4.0. The sequencing result was compared to the NCBI database, and a neighbor-joining phylogenetic tree was constructed by using the MEGA version 4.0. It was later characterized as *Bacillus amyloliquefaciens* MD4-12. A McKeen medium was used for the production of lipopeptides and consisted of (per liter of distilled water) glucose 25 g, monosodium glutamate 2.5 g, yeast extract 3.0 g, MgSO_4_·7H_2_O 1.0 g, K_2_HPO_4_ 1.0 g, KCl 0.5 g, and trace element 1.0 ml. Trace element composition (in 100 ml of distilled water) was MnSO_4_·7H_2_O 0.64 g, CuSO_4_·5H_2_O 0.16 g, and FeSO_4_·7H_2_O 0.015 g. Before sterilization, the broth pH was adjusted to 7.0. The inoculum was prepared by taking one loop of cells and inoculated into 25 mL of nutrient broth (NB) in a 250 mL Erlenmeyer flask and incubated at 30°C, 200 rpm. After overnight growth, one mL of culture was transferred to 100 mL of the production medium in a 500 mL Erlenmeyer flask and incubated in the rotary shaker at 200 rpm and 30°C for 28 h.

### 2.2. Precipitation and Extraction of Lipopeptides

The partial purification of lipopeptides was performed by precipitation and methanol extraction method [4]. Briefly, the lipopeptides were precipitated from the cell-free broth of the culture of 28 h by adjusting the supernatant pH to 2.0 using 6 N HCl and keeping it at 4°C overnight. The precipitated material was collected by centrifugation, 10.000 rpm for 15 min. This pellet was then resuspended in water by raising the pH to 7.5, lyophilized and solvent-extracted with methanol. The methanol-soluble fraction was dried using a centrifugal vacuum concentrator at 45°C. After the removal of methanol, a minimum quantity of water was added to dissolve the lipopeptides, which was again lyophilized and weighed for quantification. The crude lipopeptides were stored at −20°C for further studies.

### 2.3. Lipopeptides Characterization

The lipopeptides were characterized by UPLC-qTof-MS/MS (XEVO-G2-XS QT of waters) and fragmented for further analysis. The UPLC system used the BEH C18 reverse phase analytical column (Acquity UPLC, BEH, 1.7 *μ*m particle size, 2.1 × 50 mm, Waters). The eluent consisted of the mixture of acetonitrile and water with the ratio 40 : 60, respectively, both acidified with 0.1% formic acid with the flow rate 0.3 mL/min. Ionizations were acquired in positive ion mode using electrospray ionization mass spectrometry (ESI-MS) with capillary temperature, and the voltage was set at 300°C, 3.0 kV, respectively, and the cone voltage was 40 V. The scan ranged from *m*/*z* 50 to 2,000. The obtained data were processed by MassLynxTM software (Waters).

### 2.4. Antibacterial Activity Testing

#### 2.4.1. Bacterial Strains

Three Gram-positive reference bacteria including *S. aureus* ATCC 25923, *B. subtilis* ATCC 6633, *B. cereus* ATCC 14579 and 3 Gram-negative reference bacteria including *E. coli* ATCC 25922, *K. pneumonia* ATCC 13833, and *P. aeruginosa* ATCC 27853 were used in this study.

#### 2.4.2. Antibiotics Preparation

Stock solution of lipopeptide and ampicillin was prepared by dissolving in sterile deionized water. Aliquots of each solution were made and stored at −20°C.

#### 2.4.3. Inoculum Preparation

Inoculum was prepared as recommended by Clinical and Laboratory Standards Institute (CLSI) [20]. Bacterial strains were regenerated on NA and incubated overnight at 37°C. Two to three colonies were inoculated into Mueller–Hinton broth, and then, the turbidity was standardized to 0.5 McFarland scale (10^8^ CFU/mL). A stock culture suspension with a concentration of 10^6^ CFU/mL was made by dilutions of standardized culture and used as inoculum.

#### 2.4.4. Resazurin Preparation

Resazurin solution (0.02% w/v) was prepared by dissolving resazurin sodium salt powder in distillate water and sterilized using filter 0.22 *μ*m. The resazurin solution was kept at 4°C in a bottle and protected from the light.

#### 2.4.5. Disc Diffusion

Antimicrobial activity of the lipopeptide was performed by the disc diffusion method (Kirby–Bauer method). A sterile cotton swab was dipped into the bacterial inoculum, and the excess of the suspension was removed by pressing gently against the tube wall. The cotton swab then swabbed uniformly onto the surface of Mueller–Hinton agar (MHA) plates. Sterile Whatman no. 1 paper discs with 6 mm diameter were used and placed on the surface of an agar plate, and then, 10 *μ*L of 10 mg/mL of lipopeptide solution was impregnated into the paper disc surface. The Petri dish was incubated at 37°C for 24 hours, and the inhibition zone around the disc was measured at the end of incubation. The experiments were repeated three times, and the mean value of the inhibition zone diameter with ± standard deviation was calculated.

#### 2.4.6. Minimum Inhibitory Concentration (MIC) Determination

The MICs of lipopeptide and ampicillin were determined by resazurin microplate assay (REMA), as described by Elshikh et al. [21] with modification. The final concentration of lipopeptide and ampicillin was prepared in the range of 1024-2 *μ*g/mL. A sterile 96-well microplate with U-shaped bottom was used, and 50 *μ*L of double-strength MHB was added into well at column 1, while column 2–10 was added by 50 *μ*L of MHB. 50 *μ*L of antibiotics solution was then added to the well at column 1 and mixed gently. Two-fold serial dilutions of antibiotics were prepared by pipetting 50 *μ*L mixture from the well at column 1 and dispense to well at column 2 and so on till the last well. In the last well, 50 *μ*L of the antibiotic-MHB mixture was discarded. Column 11 used as a positive control (growth control) was filled with 50 *μ*L of double-strength MHB, and column 12 as a negative control (sterility control) was filled with 100 *μ*L MHB. Finally, 50 *μ*L of bacterial test suspension was added to each well at column 1–11 resulting in the final concentration of 5 × 10^5^ CFU/mL and then incubated for 20–22 h at 37°C. At the end of incubation, resazurin 0.02% (w/v) was added to all wells (30 *μ*l per well) except column 12 and incubated for 2 h for the observation of colour change. Columns with no colour change (blue resazurin colour remained unchanged) were scored as the MIC value.

#### 2.4.7. Synergistic Evaluation by Checkerboard Microdilution Technique

The combinations of lipopeptides and ampicillin were tested against *P. aeruginosa* ATCC 27853. The concentration of lipopeptide was ranged from 0 to 1024 *μ*g/mL while that of the ampicillin ranged from 0 to 512 *μ*g/mL. The checkerboard technique consists of the following steps: in panel B, calcium-supplemented MHB was added in all wells except rows A and H. 100 *μ*L of lipopeptide was then added into the row G (G1–G12) and row H (H1–H11; except H12) to give a final concentration of 1024 *μ*g/mL. Serial dilution from G to B was performed. In panel A, 50 *μ*L of MHB was added in all the wells except columns 1 and 12. Then, 50 *μ*L of ampicillin was added to the column 11 (A11–H11) and column 12 (A12–G12; except H12) to give a final concentration of 512 *μ*g/mL. Serial dilution from columns 11 to 2 was performed. After serially diluting panel A and panel B, 50 *μ*L was taken from each well of panel B and dispensed into the corresponding well of panel A. Then, the bacterial inoculum was prepared and added into the wells. The plates were incubated for 20–22 h at 37°C. At the end of incubation, resazurin (0.02% w/v) was added to all the wells (30 *μ*l per well) and further incubated for 2 h and for the observation of colour change.

#### 2.4.8. Fractional Inhibitory Concentration (FIC) Calculation

To evaluate the antibacterial effect of each combination, the ΣFIC was calculated:

(1) $$\begin{array}{c} \Sigma \text{FIC}=\text{FIC}\;\;\text{drug A }\left(\text{lipopeptide}\right)\;\;+\;\;\text{FIC}\;\;\text{of drug A }\left(\text{ampicillin}\right)\\ =\frac{\text{MIC}\;\;\text{of}\;\;\text{drug}\;\;\text{A}\;\;\text{in}\;\;\text{combination}}{\text{MIC }\;\;\text{of}\;\;\text{drug}\;\;\text{A}\;\;\text{alone}}+\frac{\text{MIC}\;\;\text{of}\;\;\text{drug}\;\;\text{B }\;\;\text{in}\;\;\text{combination}}{\text{MIC}\;\;\text{of}\;\;\text{drug}\;\;\text{B}\;\;\text{alone}}. \end{array}$$

The results were then classified as synergy for ΣFIC ≤ 0.5; additive for ΣFIC between 0.5 and 1.5; and indifference for values of ΣFIC between 1.5 and 2; antagonism was linked to values above 2 [22, 23].

### 2.5. Statistical Analysis

The diameters of the zones of inhibition of lipopeptides against various microbial strains were analyzed and expressed as mean values ± standard deviation. Statistical analyses were performed using one-way analysis of variance followed by the Tukey test to determine the statistically significant difference between the inhibition zone diameters of the lipopeptides against the test bacterial strains. The *p* values of less than 0.05 (*p* < 0.05) were considered significant.

## 3. Results

### 3.1. Lipopeptides Production and Extraction

*B. amyloliquefaciens* strain MD4-12 showed a direct relationship between microbial growth and lipopeptides production. During 12 hours cultivations, the exponential growth was observed, as well as the lipopeptides production (data not shown). Through medium optimization by response surface methodology, the maximum concentration of crude lipopeptides obtained was 1.25 g/L after 28 h incubation [24]. During lipopeptides extraction with methanol, yellow colour of extract solution was observed. The crude lipopeptides were stored at 4°C for further study.

### 3.2. Lipopeptides Characterization

**Figure 1:**
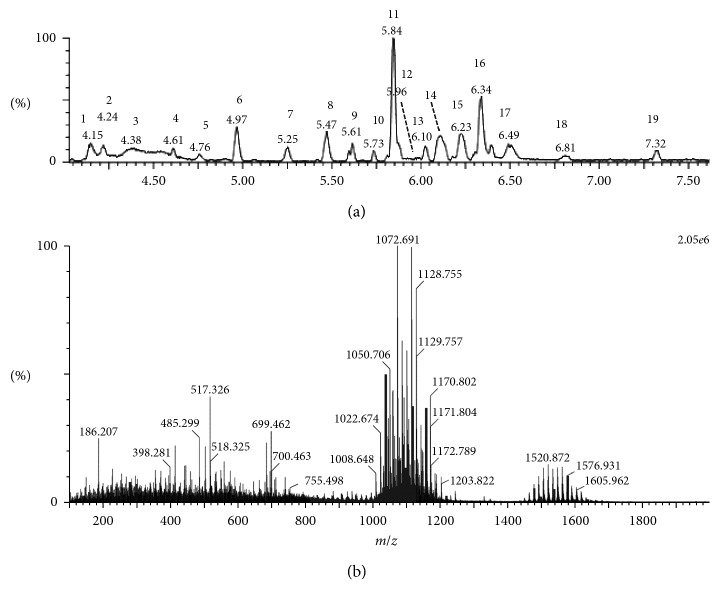
Total ion chromatogram (TIC) in the positive ion mode (a). The mass ion extracted from TIC with retention time 4.15–7.32 min (b).

**Figure 2:**
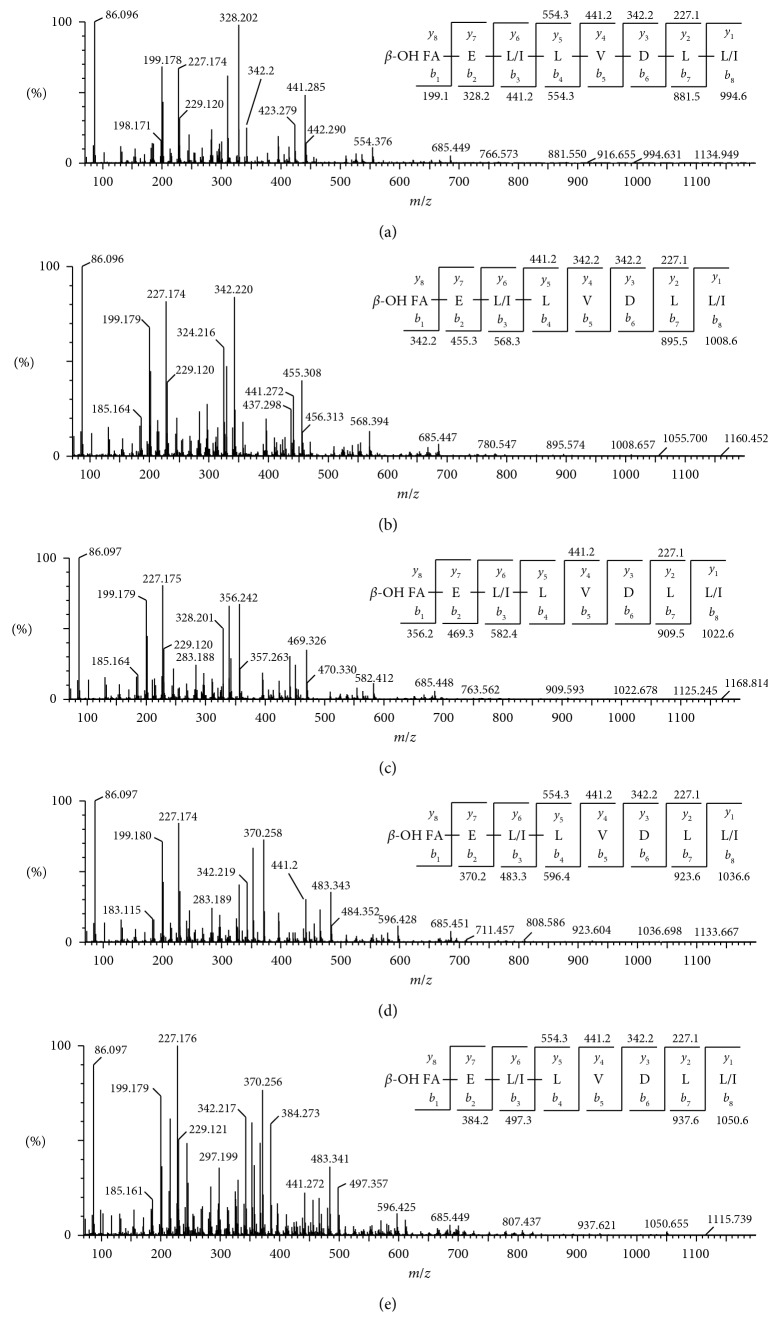
ESI-MS/MS spectra of [M + H]^+^ ions at *m*/*z* 994.62 (a), 1008.65 (b), 1022.67 (c), 1036.69 (d), and 1050.71 (e). FA, fatty acid.

ESI-MS analyses in the positive ionization mode were carried out to identify the lipopeptides produced by *B. amyloliquefaciens* strain MD4-12. The total ion chromatography (TIC) and the extracted ion chromatogram at RT 4.15–7.32 are presented in Figure 1. Nine main molecular ions in the positive ion mode were observed. Five molecular ions in the positive ion mode [M + H]^+^ with *m*/*z* 994.62, 1008.65, 1022.67, 1036.69, and 1050.71 and four in sodium ionized molecular ion [M + Na]^+^ with *m*/*z* 1031.54, 1045.60, 1059.63, and 1073.69 were observed. The molecular ion in these two group peaks differed by 14 Da from each other. Fragmentation of the mass ions was conducted only on mode [M + H]^+^ by tandem mass spectrometry and demonstrated that the five types of lipopeptides had the same peptides sequence, as presented in Figure 2. De novo peptide sequencing was conducted for assignment of peptide sequences by evaluation of *b*- and *y*-type serial ions. This revealed that the five types of lipopeptides had the same sequence with surfactin homologs [25–27]. The assignments of the entire ion mass are presented in Table 1.

### 3.3. Antimicrobial Activity Test

The results of the antimicrobial activity of the lipopeptide against several bacteria tests are presented in Table 2. The lipopeptide showed activity against Gram-positive as well as Gram-negative bacterial strains with varying inhibition zone diameters recorded. The lowest inhibition zone of 9.26 ± 0.65 mm displayed against *B. subtilis* ATCC 6633 and the highest against *S. aureus* ATCC 25923 at 20.0 ± 0.53 mm (Table 1). These biological properties were important to describe potential applications of the lipopeptide, especially in the biomedical field.

### 3.4. Synergistic Evaluation

In this research, a combinatorial effect of surfactin and ampicillin was evaluated. MIC was determined by the broth microdilution method because it only uses a small quantity of samples.  Table 3 shows MIC of surfactin, either alone or in combination with ampicillin against *P. aeruginosa* ATCC 27853. Surfactin alone had little activity, with MIC >1024 *μ*g/mL, while ampicillin alone had MIC 256 *μ*g/mL showed that ampicillin was not effective to inhibit *P. aeruginosa* ATCC 27853. A similar result of ampicillin activity was reported by Reimer et al. [28]. Analysis combination between surfactin and ampicillin at several concentrations by the checkerboard microdilution method revealed greater antibacterial effect than that observed alone. Combination of surfactin in the range from 32 to 512 *μ*g/mL with ampicillin 64 *μ*g/mL showed synergistic effect with FIC 0.28–0.5.

## 4. Discussion

Lipopeptides analysis by ESI-MS yielded the main ions with *m*/*z* 994.62, 1008.65, 1022.67, 1036.69, and 1050.71. Fragmentation of these main ions resulted in *b*- and *y*-type serial fragment ions, as shown in Figure 2. The use of electron spray for surfactin ionization by mass spectrometry was reported often to result in sodium adduct that arises together with other main ions as minor peaks [29]. The free carboxylic acid group in surfactin binds to the alkali metal ions and form metal ion adducts. Sodium and potassium adducts of surfactin are the most frequently observed since these metal ions are ubiquitously present in nature [30]. The difference in mass of 14 Da of the precursor ions showed that surfactin varied only in the fatty acid moiety that was composed of *β*-hydroxy fatty acids of varying lengths: C12 (*m*/*z* 994), C13 (*m*/*z* 1008), C14 (*m*/*z* 1022), C15 (*m*/*z* 1036), and C16 (*m/z* 1050).

The marker ions of surfactin with *m/z* 441 and 685 were also observed in every fragmentation of each parent ion as the product of the cleavage between Glu-Leu and FA-Leu, with the net charge retained in the residual hexapeptide (Leu-Leu-Val-Asp-Leu-Leu) and the product of an y6-b5 cleavage that yields the residual tetrapeptide (Leu-Leu-Val-Asp), respectively [30]. The fragment ions with 18 Da and 28 Da difference mass respective to parent ions were also observed. For example, fragmentation of the precursor ion with *m*/*z* 1036 yielded ion peak with *m*/*z* 465 (18 Da mass differences to its parent ion with *m/z* 483; b2-H_2_O). This ion peak corresponds to a loss of H_2_O from its parent ion. In addition, fragment ions with *m*/*z* 199 (a1) and 328 (a2) were also observed as result from the loss of carbonyl mass (-C=O; 28 Da) from its parent ions *m*/*z* 227 (*y*2) and 328 (*b*1), respectively (Figure 2). Those ion peaks seem to be a general feature during the fragmentation of cyclic depsipeptides [29]. Therefore, by *de novo* sequencing, based on each serial fragment ion revealed that the peptide moiety of surfactin produced by *B. amyloliquefaciens* strain MD4-12 has the same amino acid sequence, consisting of Glu-Leu/Ile-Leu-Val-Asp-Leu-Leu/Ile.

Surfactin is synthesized nonribosomally in *Bacillus* that involves multienzymes called nonribosomal peptide synthetases [31]. The surfactin structure consists of a cyclic peptide linked to a fatty acid moiety. The primary surfactin molecule contains the heptapeptide sequence Glu-Leu-Leu-Val-Asp-Leu-Leu and C_12_-C_17_*β*-hydroxy fatty acid chain. The lipopeptides belonging to surfactin family have several variants which may differ in amino acid composition (Ala, Val, Leu, or Ile amino acid variations at positions 2, 4, and 7) or differing in the length, branching, and saturation of their acyl chain [26, 32, 33]. Variation of surfactin is highly dependent on strain, culture condition, and growth medium composition [36]. Structural diversity of surfactin can influence its physiochemical properties and biological activities significantly including interaction with the microbial membrane [25, 34]. Surfactin has been reported having activity against Gram-positive as well as Gram-negative bacteria [35, 37]. The mechanism action of the lipopeptides has also been studied [38]. There are three hypotheses that describe how surfactin work against Gram-negative bacteria. (a) Cation scavenging, lipopeptides works by removing Ca and Mg from liposaccharides (LPS) causing the outer membrane destabilization. Lipopeptides can also bind Ca and Mg effectively, so that the *P. aeruginosa* membrane is permeable to beta-lactam antibiotics. (b) Pore-forming effect. The pore-forming (ion channel) effect on the membrane cell is characterized by the formation of cationic channels. The pore-forming was explained by several models, namely, carpet, barrel stave, and toroidal models. (c) Detergent effect. Detergent effect refers to the ability of surfactin to insert a chain of fatty acids into the bilipidic layer, causing disorganization that causes the membrane to be permeable [38]. Insertion of several surfactin molecules into the membrane can form mixed micelles through self-association and bilayer mechanisms by the hydrophobic fatty acid chain which eventually leads to the solubilization of bilayers [39]. The appropriateness of each mechanism depends on the peptide, as well as properties of the lipids (i.e., phase, elasticity, hydrophobic chain length, and hydration) [40].

*P. aeruginosa* is a pathogen that shows resistance to many antimicrobial agents. Part of the resistance mechanisms is indicated by low permeability of the outer membrane, type 1 AmpC *β*-lactamase expression, and multidrug efflux systems [39]. Those resistance systems causing many beta-lactam antibiotics were not effective to kill *P. aeruginosa*. The usage of antibiotic combination for therapy can limit and suppresses bacterial resistance, decreases antibiotic toxicity, covers a broad range of pathogens with greater efficacy, and most importantly, leads to synergy [22, 23].

A synergistic effect can occur through various mechanisms. Synergistic antibacterial effect is usually often observed when between the compounds that work at different targets. A possible explanation of the synergistic effect between lipopeptides and ampicillin is that the lipopeptide damages the cell wall sufficiently to increase ampicillin entry. It promotes ampicillin action to inhibit transpeptidase that catalyzes cross-linking of peptidoglycan in cell wall biosynthesis.

## 5. Conclusion

*B. amyloliquefaciens* strain MD4-12 isolated from oil-contaminated soil has the ability to produce lipopeptide when cultivated using the McKeen medium. By ESI-MS, the lipopeptide could be identified putatively as surfactin. Further analysis using tandem mass spectrometry, six variants of surfactin (C_12_ to C_17_ surfactin) with the same amino acid sequence in peptide moiety could be revealed. Surfactin showed low activity against Gram-negative bacteria of *P. aeruginosa*. Combination surfactin with ampicillin against *P. aeruginosa* showed a synergistic effect.

## Figures and Tables

**Table 1:** Peak numbers, retention times, mass ion peaks, and assignment of the fatty acid moiety of surfactin.

Peak number	UPLC retention time (min)	Mass peak (*m*/*z*)		Assignment
		[M + H]^+^	[M + Na]^+^	
11	5.84	994.62		C_12_-surfactin
12; 13; 15	5.96; 6.10; 6.23	1008.65	1031.54	C_13_-surfactin
12; 16	6.10; 6.34	1022.67	1045.60	C_14_-surfactin
11; 2; 4; 5; 6; 7; 8; 9; 10; 15	4.15; 4.24; 4.61; 4.76; 4.97; 5.25; 5.47; 5.61; 5.73; 6.23	1036.69	1059.63	C_15_-surfactin
1; 16	4.15; 6.34	1050.71	1073.69	C_16_-surfactin

**Table 2:** Inhibition zone diameter of lipopeptides against various reference bacterial strains. Results are expressed as the mean value of three independent experiments ± standard deviations.

Microorganism	Inhibition zone diameter (mm) ± SD
*S. aureus* ATCC 25923	20.0 ± 0.5
*B. subtilis* ATCC 6633	9.3 ± 0.7
*B. cereus* ATCC 14579	13.5 ± 1.5
*E. coli* ATCC 25922	11.4 ± 0.6
*K. pneumonia* ATCC 13833	13.9 ± 0.4
*P. aeruginosa* ATCC 27853	15.7 ± 0.7

**Table 3:** MIC of SRF and AMP used alone and in combination against *P. aeruginosa* ATCC 27853.

Antimicrobial	MIC (*μ*g/mL)		FIC	Effect
	Alone	In combination		
SRF, AMP	>1024, 256	16, 128	0.52	Additive
SRF, AMP	>1024, 256	32, 64	0.28	Synergy
SRF, AMP	>1024, 256	64, 64	0.31	Synergy
SRF, AMP	>1024, 256	128, 64	0.38	Synergy
SRF, AMP	>1024, 256	256, 64	0.50	Synergy
SRF, AMP	>1024, 256	512, 64	0.63	Additive

SRF, surfactin; AMP, ampicillin.

## Data Availability

The data used to support the findings of this study are included within the article.
